# Supplementary material for: Specificity and sensitivity of an RNA targeting type III CRISPR complex coupled with a NucC endonuclease effector
Source: Nucleic Acids Res. 2021 Dec 6;49(22):13122–34. doi: 10.1093/nar/gkab1190 (PMC8682760; doi:10.1093/nar/gkab1190)
Supplement: gkab1190_Supplemental_File [file gkab1190_supplemental_file.pdf]

SUPPORTING INFORMATION

Specificity and sensitivity of an RNA targeting type III CRISPR complex coupled with a NucC endonuclease effector

*Sabine Grüschow, Catherine S Adamson and Malcolm F. White**

Biomedical Sciences Research Complex, School of Biology, University of St Andrews, St Andrews, KY16 9ST, UK

*To whom correspondence should be addressed: Tel +44-1334 463432; Fax +44-1334462595; email mfw2@st-andrews.ac.uk;

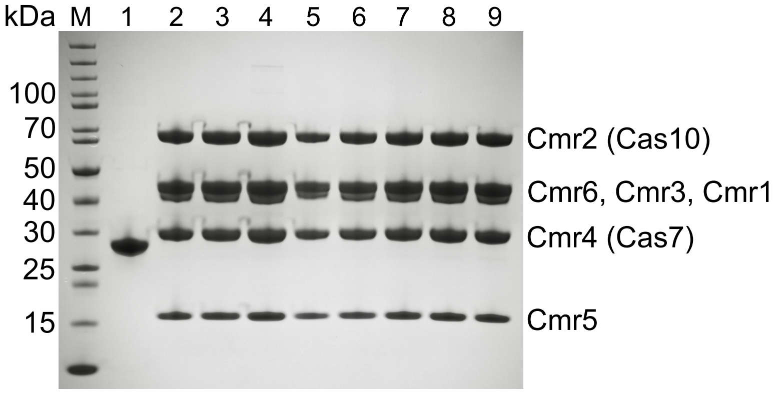

Figure S1.SDS-PAGE of purified VmeCmr complexes and VmeNucC used in assays. M: PageRuler Unstained (Thermo Scientific) protein marker; 1: VmeNucC; 2: VmeCmr^{pUC}; 3: VmeCmr^{N57}; 4: VmeCmr^{N209}; 5: VmeCmr^{N209} Cmr4 D26A; 6: VmeCmr^{N320}; 7: VmeCmr^{N719}; 8: VmeCmr^{N782}; 9: VmeCmr^{N1169}. The crRNA target for each Cmr complex is indicated as superscript.

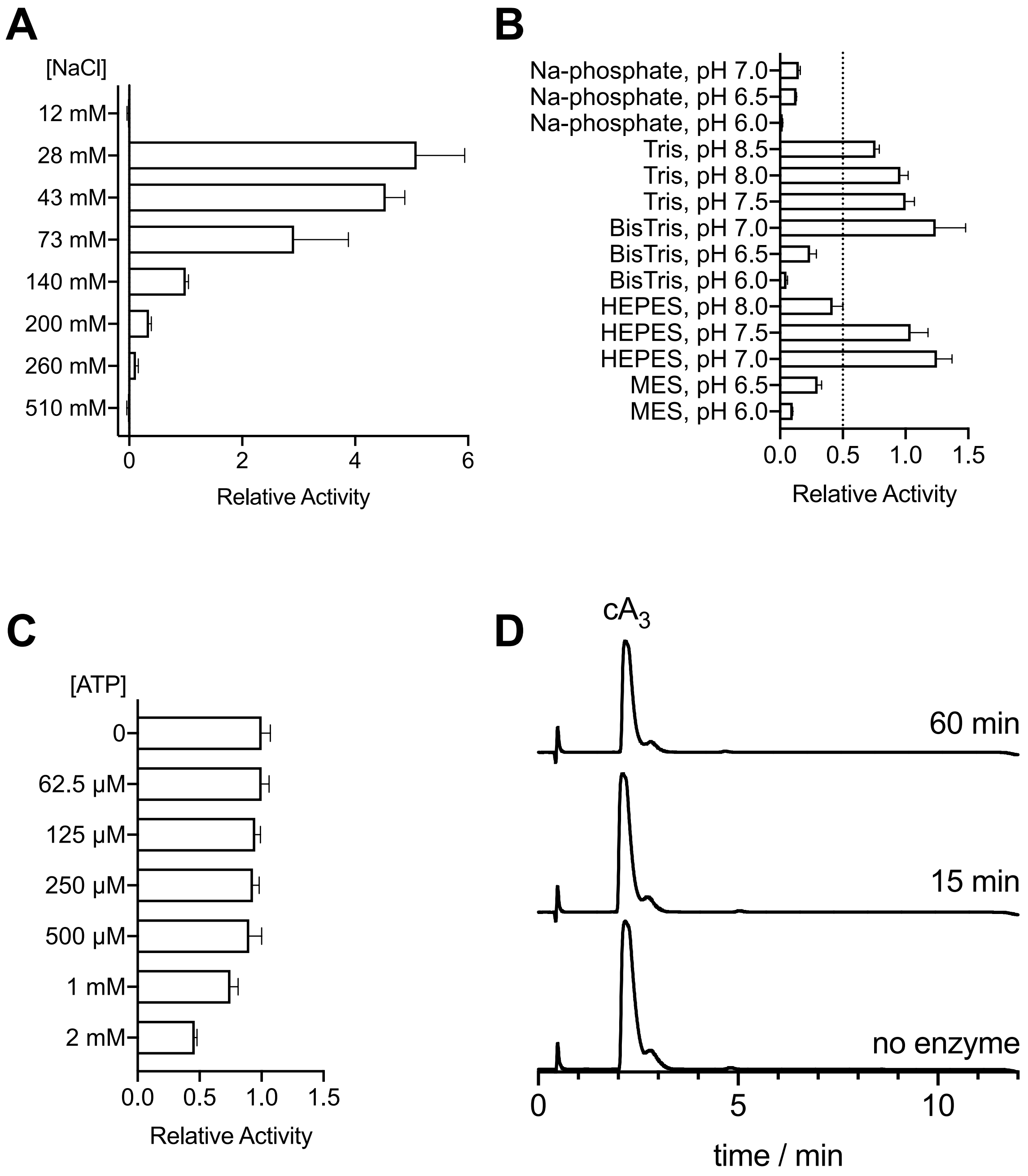

Figure S2.Factors influencing NucC activity. Relative activities were determined by comparing the rates of the reactions relative to an arbitrarily chosen reference condition. Rates were assumed to be proportional to the change in fluorescence signal over time and calculated from the slopes of linear regression analysis. A: Effect of NaCl concentration. B: NucC activity in different buffers. C: Effect of ATP on NucC nuclease activity. D: LC chromatograms with UV monitoring at 254 nm showing the reaction of 100 μM cA_3 with 0.5 μM NucC under assay conditions after 15 and 60 min. A reaction in which NucC had been omitted was allowed to proceed for 60 min.

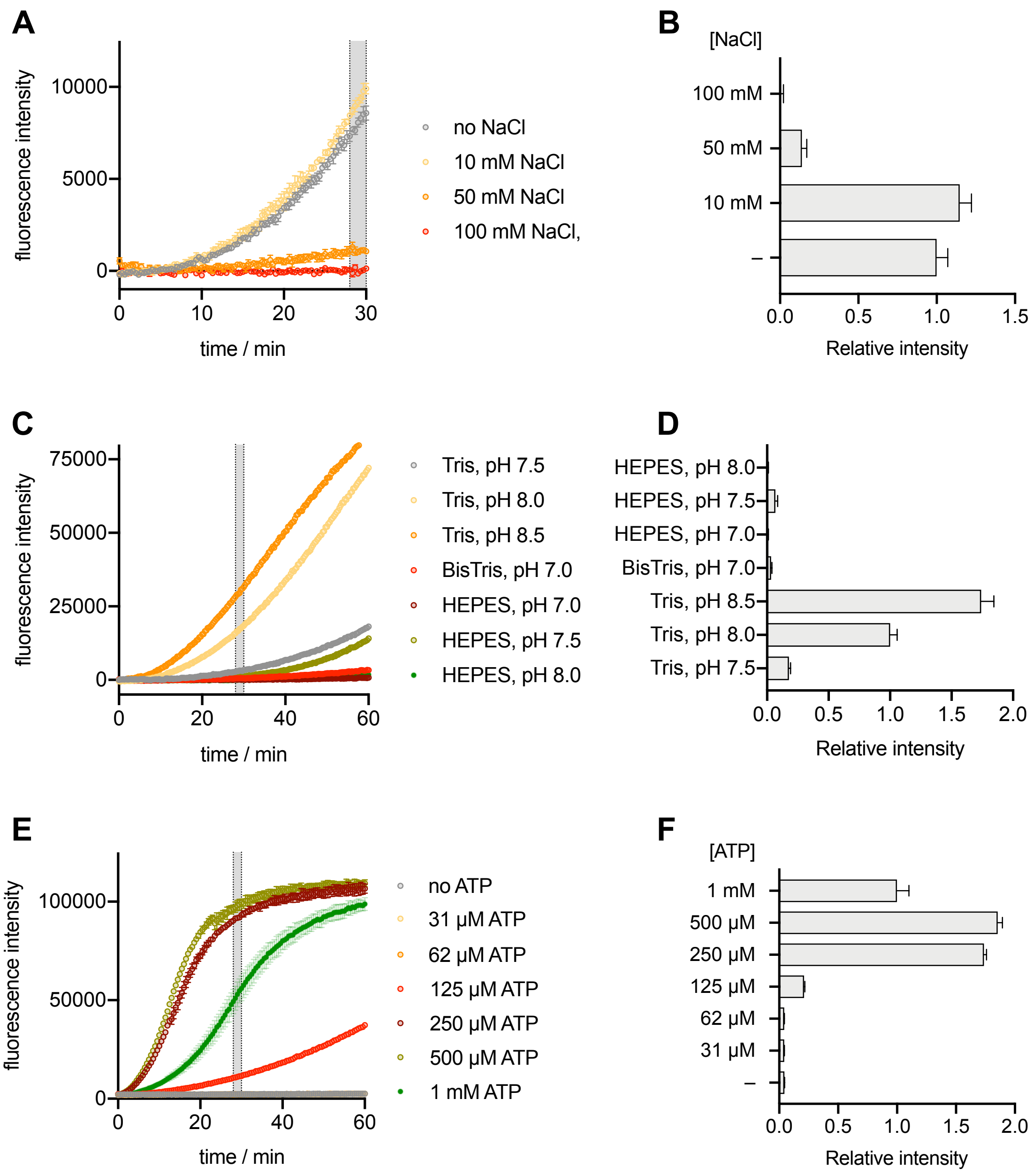

Figure S3. Screening of reaction conditions for the coupled VmeCmr/NucC assay. A, C, E: Progress curves. B, D, F: Fluorescence intensities extracted from corresponding progress curves (28 – 30 min, shaded in grey) relative to an arbitrarily chosen reference condition. A and C were baseline corrected using the progress curves obtained in the absence of target; no baseline correction was performed for E. Reaction conditions for A and B: 100 nM Cmr^{pUC}, 200 pM pUC target, 50 mM Tris-HCl (pH 7.5), 6 % (v/v) glycerol, 10 mM $MgCl_2$, 250 µM ATP, 12 mM NaCl. Additional NaCl was supplemented as indicated in the graphs; C and D: 100 nM Cmr^{pUC}, 200 pM pUC target, 50 mM buffer as indicated, 22 mM NaCl, 6 % glycerol, 10 mM $MgCl_2$, 250 µM ATP; E and F: 100 nM Cmr^{pUC}, 200 pM pUC target, 12.5 mM HEPES (pH 7.5), 12 mM NaCl, 6 % glycerol, 10 mM $MgCl_2$, ATP as indicated. All reactions contained 250 nM NucC and 125 nM NucC substrate. They were preincubated for 10 min at 37 °C before addition of NucC. Assays were performed in duplicate

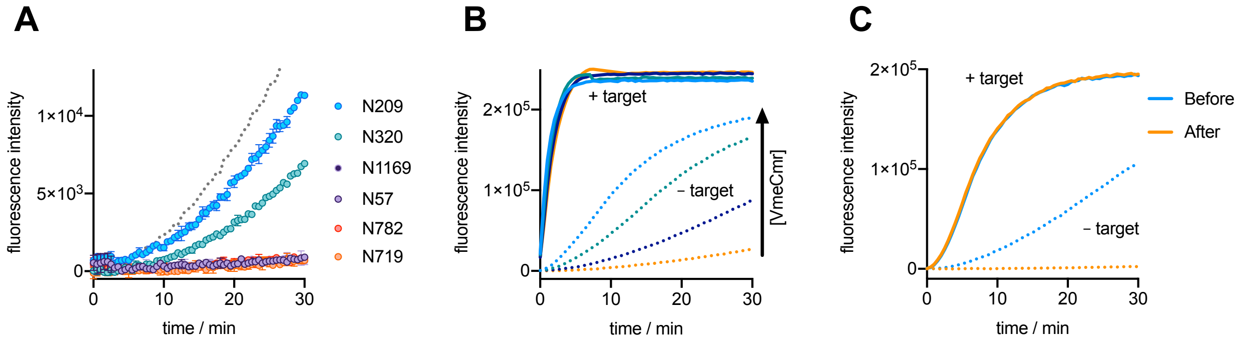

Figure S4. Factors influencing unregulated cA_3 synthesis in VmeCmr complexes. Progress curves of the coupled VmeCmr/NucC assay are shown. Only the mean of duplicate reactions is shown for clarity (B, C). N gene transcript was used as target RNA. **A**: VmeCmr / NucC background activity in the absence of target RNA. Companion graph to Figure 6B. The dotted curve corresponds to N782 in the presence of 2.6 pM target RNA (provided for scale). **B**: Reducing the VmeCmr concentration can reduce background activity (in the absence of target RNA) without affecting target-induced cA_3 synthesis. $VmrCmr^{N209}$ before heparin purification was used at 31 – 250 nM. **C**: Further purification by heparin chromatography significantly reduced the background activity without affecting target-induced activity (100 nM $VmeCmr^{N320}$).

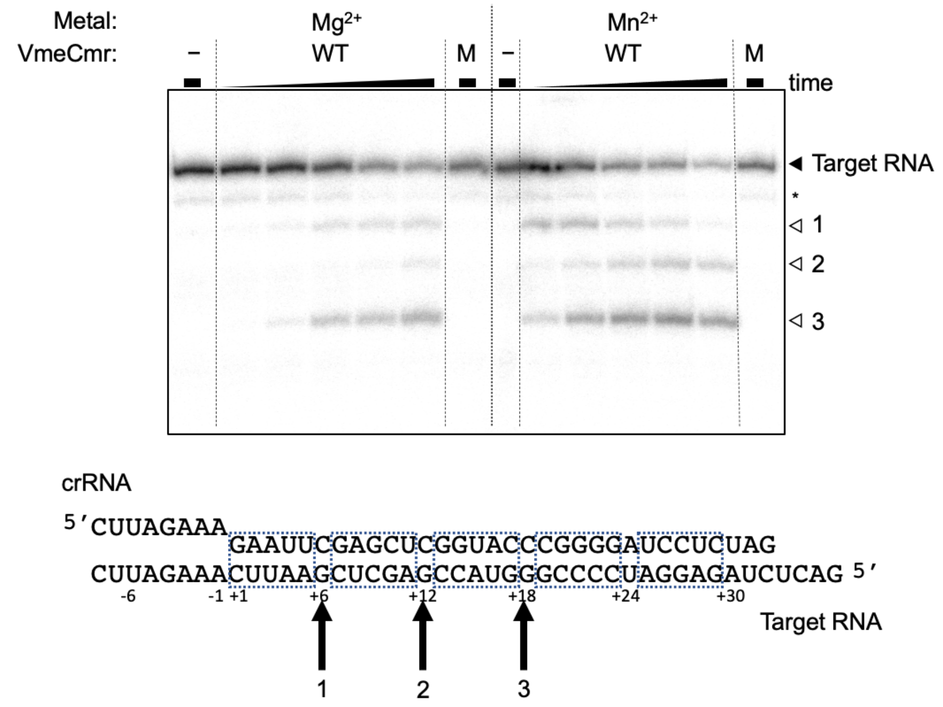

Figure S5. Denaturing polyacrylamide gel analysis of target RNA cleavage by VmeCmr^{pUC}. The reaction was performed under standard conditions with either 100 nM wild type (WT) or Cmr4$_{D26A}$ mutant (M) and 1 nM 5'-^{32}P-labeled target RNA and quenched by addition of 50 mM EDTA followed by phenol:chloroform extraction after 2, 5, 10, 30, and 60 min. Approximately 50 % target RNA was cleaved after 1 h incubation with either Mg^{2+} or Mn^{2+} as metal co-factor. The Cmr4$_{D26A}$ mutant is devoid of RNase activity in the presence of either metal ion. The asterisk indicates a contaminant from the target RNA. Three cleavage sites are indicated by open arrows labelled 1-3. The sequence of the crRNA:target RNA duplex is shown below, with the potential cleavage sites 1-3 indicated by arrows. The position of the three cleavage sites (+6, +12 and +18) is estimated based on gel migration position with respect to the loading dyes and uncut RNA. The gel is representative of two independent experiments.

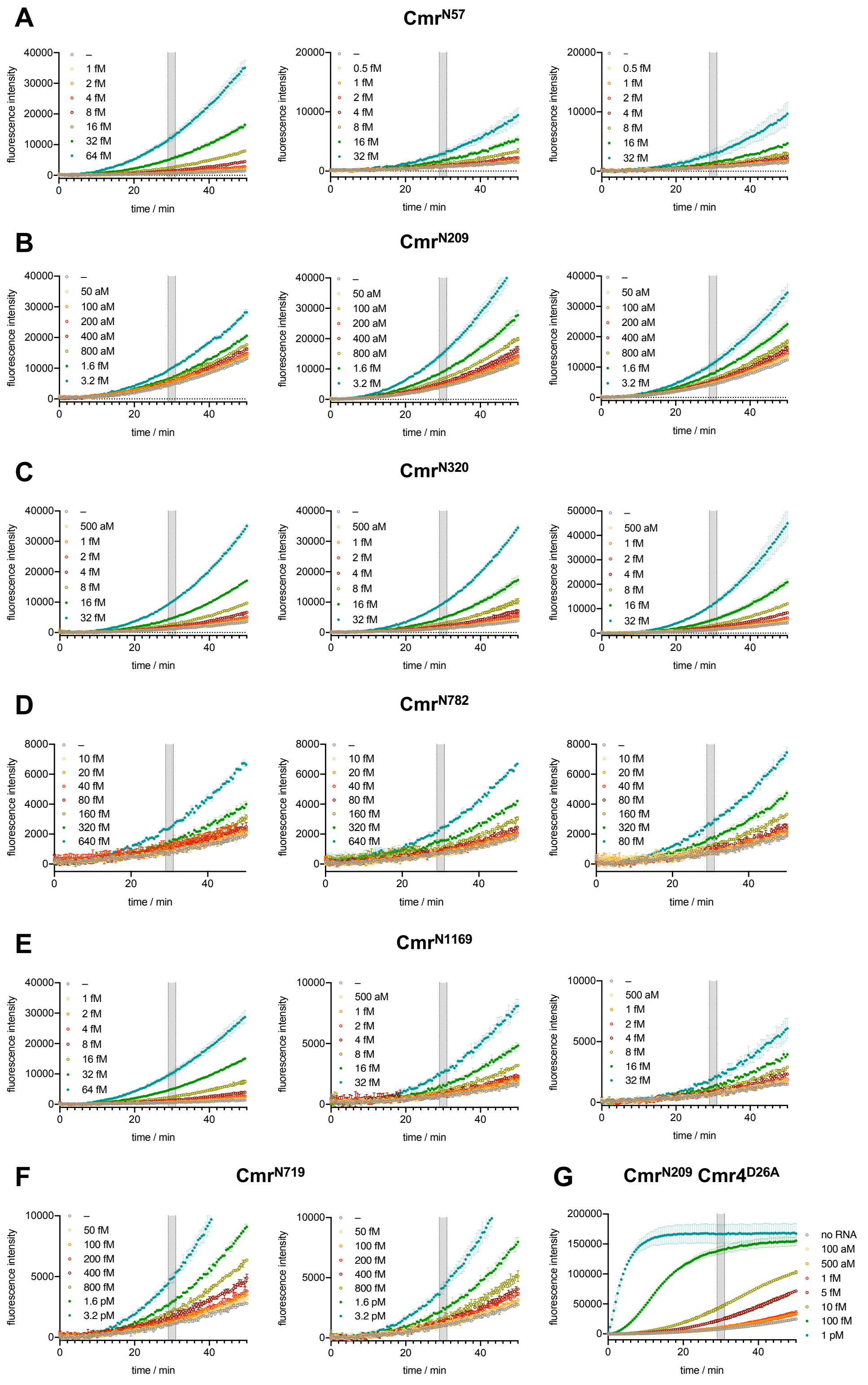

Figure S6. Progress curves used to determine the LoD of SARS-CoV-2-targeting VmeCmr complexes. The mean and standard deviation of the fluorescence intensities are shown. Standard assay conditions as described in Materials and Methods were used. The Cmr concentrations were 100 nM for A (Cmr^{N57}), D (Cmr^{N782}), E (Cmr^{N1169}), and F (Cmr^{N719}), 50 nM for B (Cmr^{N209}), and C (Cmr^{N320}), 25 nM for G (Cmr^{N209} Cmr4 D26A). N gene transcript at indicated concentration ranges was used as target throughout. The fluorescence intensities between 29 – 31 min (shaded in grey, Figure S) were used to determine the LoD. Each independent experiment was performed in duplicate.

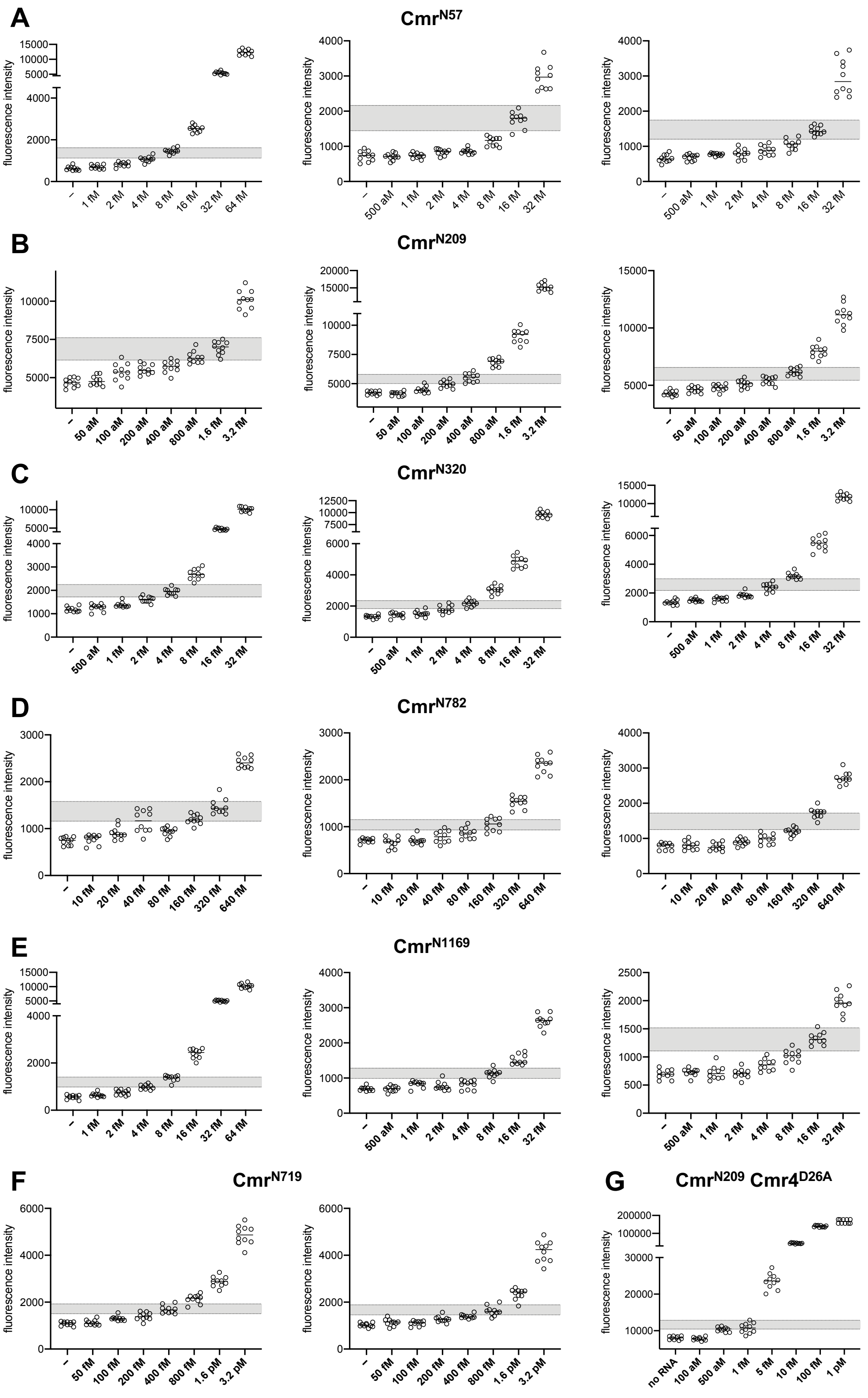

Figure S7. Fluorescence intensities used to determine the LoD of SARS-CoV-2-targeting VmeCmr complexes. The fluorescence intensities between 29 – 31 min are shown as individual values and mean. The shaded area corresponds to *y* values between 5 and 10 standard deviations greater than the mean of the reference (no RNA). The lowest measured target concentration (mean) above the shaded area was taken as the LoD. For clarity, the *y* axis has been split for some experiments and the scaling of the two sections is not identical.

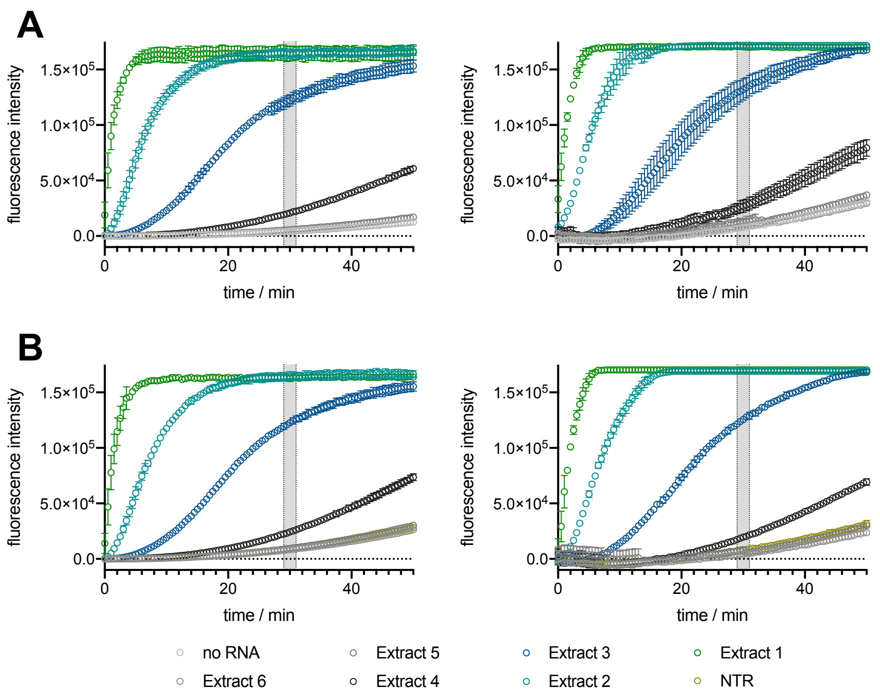

Figure S8. Detection of SARS-CoV-2 N gene in viral extracts. Extracts 1 – 6 were obtained by extracting a 10-fold serial dilution of viral stocks ranging from $6 \cdot 10^6$ to $6 \cdot 10^1$ PFU ml^{-1}, respectively. The assay was performed under standard conditions in triplicate. **A**: 50 nM wild type $VmeCmr^{N209}$; **B**: 25 nM $VmeCmr^{N209}$ Cmr4 D26A. NTR: non-target RNA. Complete data set for Figure 8. Two different batches of SARS-CoV-2 RNA were used in each set.

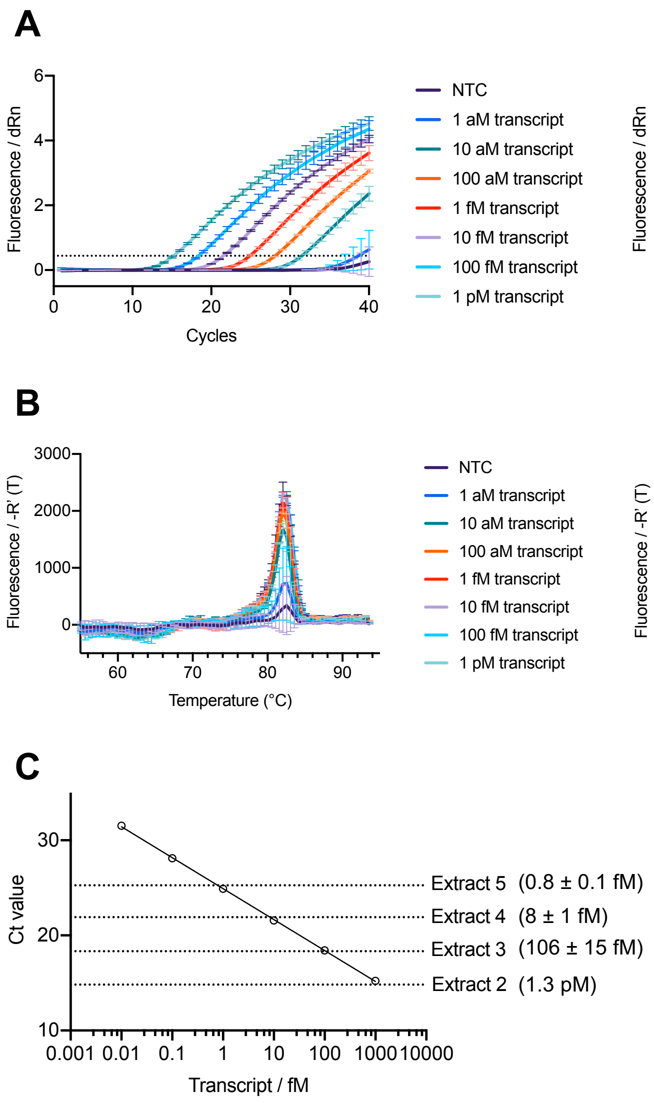

Figure S9. RT-qPCR analysis of SARS-CoV-2 N gene transcript and genomic RNA extracts. A: Amplification plots, the threshold value is indicated by a dotted line; **B**: dissociation curves; **C**: standard curve based on transcript dilution series (10 aM – 1 pM, R^2 0.9999), the Ct values of the extracts are indicated by dotted lines; the concentrations were calculated from non-linear regression (semi-log line, Prism). NTC: no template control; NRT: no reverse transcriptase control. No DNA contamination was detected in the tested SARS-CoV-2 genomic RNA extracts. Only the highest N gene transcript concentration was tested for DNA contamination; even though detectable levels of DNA were present in this sample, most likely through incomplete DNA digestion during work-up of the *in vitro* generated transcript, it was deemed negligible for the purpose of RNA quantitation (ΔCt = -18.4 corresponding to $10^5 – 10^6$-fold excess of RNA over DNA).

Table S1. Data sets used for determination of LoDs.

**pUC (1)**	no RNA	NTR	78.1 fM	156.3 fM	312.5 fM	625 fM	1.25 pM	2.5 pM
mean (N = 10)	15073	15599	20414	22805	30846	48161	72971	92436
SD	832	855	1145	1266	1483	1787	1668	1254
Threshold	23395	24151						
Sample above threshold?	Ref		nd	nd	detected	detected	detected	detected
		Ref	nd	nd	detected	detected	detected	detected
**pUC (1)**	no RNA	NTR	78.1 fM	156.3 fM	312.5 fM	625 fM	1.25 pM	2.5 pM
mean (N = 10)	13455	14689	15338	15568	21071	31320	47989	66849
SD	1078	988	823	924	1213	2015	2830	3270
Threshold	24233	24569						
Sample above threshold?	Ref		nd	nd	nd	detected	detected	detected
		Ref	nd	nd	nd	detected	detected	detected
**N57 (1)**	no RNA	1 fM	2 fM	4 fM	8 fM	16 fM	32 fM	64 fM
mean (N = 10)	629	705	826	1070	1467	2529	5434	12340
SD	99	95	106	134	123	164	459	952
Threshold	1617							
Sample above threshold?		nd	nd	nd	nd	detected	detected	detected
**N57 (2)**	no RNA	500 aM	1 fM	2 fM	4 fM	8 fM	16 fM	32 fM
mean (N = 10)	724	708	735	839	856	1135	1757	2965
SD	144	95	75	85	73	128	224	352
Threshold	2162							
Sample above threshold?		nd	nd	nd	nd	nd	nd	detected
**N57 (3)**	no RNA	500 aM	1 fM	2 fM	4 fM	8 fM	16 fM	32 fM
mean (N = 10)	656	686	770	789	883	1058	1452	2968
SD	109	86	35	139	140	152	120	517
Threshold	1746							
Sample above threshold?		nd	nd	nd	nd	nd	nd	detected
**N209 (1)**	no RNA	50 aM	100 aM	200 aM	400 aM	800 aM	1.6 fM	3.2 fM
mean (N = 10)	4680	4802	5373	5543	5723	6321	6958	10096
SD	293	315	568	315	387	392	428	623
Threshold	7613							
Sample above threshold?		nd	nd	nd	nd	nd	nd	detected
**N209 (2)**	no RNA	50 aM	100 aM	200 aM	400 aM	800 aM	1.6 fM	3.2 fM
mean (N = 10)	4214	4137	4499	4961	5522	6839	9089	15256
SD	158	178	273	296	375	319	575	1091
Threshold	5793							
Sample above threshold?		nd	nd	nd	nd	detected	detected	detected
**N209 (3)**	no RNA	50 aM	100 aM	200 aM	400 aM	800 aM	1.6 fM	3.2 fM
mean (N = 10)	4305	4617	4798	5116	5405	6180	7968	11176
SD	225	254	284	344	336	322	540	886
Threshold	6557							
Sample above threshold?		nd	nd	nd	nd	nd	detected	detected
**N209 Cmr4^{D26A}**	no RNA	100 aM	500 aM	1 fM	5 fM	10 fM	100 fM	1 pM
mean (N = 10)	8021	7793	10357	10717	23527	44994	139748	166704
SD	482	450	608	1378	2134	2922	4861	11507
Threshold	12845							
Sample above threshold?		nd	nd	nd	detected	detected	detected	detected
**N320 (1)**	no RNA	500 aM	1 fM	2 fM	4 fM	8 fM	16 fM	32 fM
mean (N = 10)	1187	1267	1376	1611	1946	2693	4733	10115
SD	106	149	110	125	140	226	297	650
Threshold	2249							
Sample above threshold?		nd	nd	nd	nd	detected	detected	detected
**N320 (2)**	no RNA	500 aM	1 fM	2 fM	4 fM	8 fM	16 fM	32 fM
mean (N = 10)	1320	1426	1525	1810	2179	3053	4840	9625
SD	103	150	183	244	204	252	358	642
Threshold	2346							
Sample above threshold?		nd	nd	nd	nd	detected	detected	detected
**N320 (3)**	no RNA	500 aM	1 fM	2 fM	4 fM	8 fM	16 fM	32 fM
mean (N = 10)	1358	1497	1572	1851	2401	3186	5460	11788
SD	163	106	134	174	277	222	467	873
Threshold	2990							
Sample above threshold?		nd	nd	nd	nd	detected	detected	detected

Table S1 cont.

N719 (1)	no RNA	10 fM	20 fM	40 fM	80 fM	160 fM	320 fM	640 fM
mean (N = 10)	990	1022	1160	1092	1162	1151	1288	1507
SD	106	114	97	113	132	153	139	114
Threshold	2050							
Sample above threshold?		nd	nd	nd	nd	nd	nd	nd
**N719 (2)**	no RNA	50 fM	100 fM	200 fM	400 fM	800 fM	1.6 pM	3.2 pM
mean (N = 10)	1088	1128	1306	1394	1690	2135	2881	4862
SD	83	113	96	163	168	185	219	413
Threshold	1923							
Sample above threshold?		nd	nd	nd	nd	detected	detected	detected
**N719 (3)**	no RNA	50 fM	100 fM	200 fM	400 fM	800 fM	1.6 pM	3.2 pM
mean (N = 10)	1027	1137	1100	1275	1392	1625	2347	4158
SD	86	147	104	131	84	208	229	451
Threshold	1884							
Sample above threshold?		nd	nd	nd	nd	nd	detected	detected
**N782 (1)**	no RNA	10 fM	20 fM	40 fM	80 fM	160 fM	320 fM	640 fM
mean (N = 10)	737	773	910	1156	938	1197	1468	2412
SD	84	100	132	237	89	99	162	121
Threshold	1579							
Sample above threshold?		nd	nd	nd	nd	nd	nd	detected
**N782 (2)**	no RNA	10 fM	20 fM	40 fM	80 fM	160 fM	320 fM	640 fM
mean (N = 10)	708	657	708	787	864	1045	1522	2328
SD	44	111	83	126	114	134	119	180
Threshold	1151							
Sample above threshold?		nd	nd	nd	nd	nd	detected	detected
**N782 (3)**	no RNA	10 fM	20 fM	40 fM	80 fM	160 fM	320 fM	640 fM
mean (N = 10)	780	808	765	900	981	1194	1719	2721
SD	94	124	108	95	139	106	147	173
Threshold	1719							
Sample above threshold?		nd	nd	nd	nd	nd	nd	detected
**N1169 (1)**	no RNA	1 fM	2 fM	4 fM	8 fM	16 fM	32 fM	64 fM
mean (N = 10)	559	641	753	984	1360	2399	5015	10250
SD	84	84	115	100	126	203	265	852
Threshold	1403							
Sample above threshold?		nd	nd	nd	nd	detected	detected	detected
**N1169 (2)**	no RNA	500 aM	1 fM	2 fM	4 fM	8 fM	16 fM	32 fM
mean (N = 10)	694	692	827	771	812	1130	1522	2632
SD	59	78	91	123	128	121	140	181
Threshold	1280							
Sample above threshold?		nd	nd	nd	nd	nd	detected	detected
**N1169 (3)**	no RNA	500 aM	1 fM	2 fM	4 fM	8 fM	16 fM	32 fM
mean (N = 10)	694	727	721	706	860	1005	1331	1975
SD	82	66	121	86	104	127	107	182
Threshold	1517							
Sample above threshold?		nd	nd	nd	nd	nd	nd	detected
**N209 (1)**	no RNA	Extract 6	Extract 5	Extract 4	Extract 3	Extract 2	Extract 1	SARS-Cov-2
mean (N = 15)	4432	4281	5717	20841	122614	164411	164551	
SD	385	381	345	1652	4955	3716	5617	
Threshold	8281							
Sample above threshold?		nd	nd	detected	detected	detected	detected	
**N209 (2)**	no RNA	Extract 1	Extract 2	Extract 3	Extract 4	Extract 5	Extract 6	SARS-Cov-2
mean (N = 15)	7537	170419	171694	132152	27056	12308	8939	
SD	782	1788	1979	8468	4743	3676	2323	
Threshold	15360							
Sample above threshold?		detected	detected	detected	detected	nd	nd	

The tested VmeCmr complexes are indicated by their crRNA target. The threshold was defined as the reference mean plus 10 reference standard deviations. If the sample mean was above the threshold, the sample was deemed “detected”, and “nd” (not detected) if it was below.

Table S2. Ct values from RT-qPCR analysis.

**1 aM** [a)]	**10 aM** [a)]	**100 aM** [a)]	**1 fM** [a)]	**10 fM** [a)]	**100 fM** [a)]	**1 pM** [a)]
36.7	31.29	28.14	24.78	21.67	18.28	15.06
No Ct	31.53	28.07	24.92	21.57	18.41	15.36
38.71	31.76	28.13	25.01	21.51	18.58	15.23
						**NRT**
						33.74
						33.53
						34.08

**NTC**	**Extract 1** [b)]	**Extract 2** [b)]	**Extract 3** [b)]	**Extract 4** [b)]
38.34	14.74	18.24	21.90	25.15
No Ct	14.96	18.33	22.00	25.35
No Ct	14.75	18.42	21.83	25.29
		**NRT**	**NRT**	**NRT**
		No Ct	No Ct	No Ct
		No Ct	No Ct	No Ct
		No Ct	No Ct	No Ct

a) SARS-CoV-2 N gene transcript
b) SARS-CoV-2 genomic RNA extract
NTC: no template control; NRT: no reverse transcriptase control
